# Reflux episodes and esophageal impedance levels in patients with typical and atypical symptoms of gastroesophageal reflux disease

**DOI:** 10.1097/MD.0000000000007978

**Published:** 2017-09-15

**Authors:** Bi Xing Ye, Liu Qin Jiang, Lin Lin, Ying Wang, Meifeng Wang

**Affiliations:** a Department of Gastroenterology, The First Affiliated Hospital of Nanjing Medical University; b Department of Gastroenterology, Jiangsu Province Official Hospital, Nanjing, Jiangsu, China.

**Keywords:** acid reflux, atypical symptoms, mean nocturnal baseline impedance, nonacid reflux, typical symptoms

## Abstract

To determine the relationship between baseline impedance levels and gastroesophageal reflux, we retrospectively enrolled 110 patients (54 men and 56 female; mean age, 51 ± 14 years) with suspected gastroesophageal reflux disease (GERD) who underwent 24-h multichannel intraluminal impedance and pH monitoring. Patients were stratified according to symptom (typical or atypical) and reflux types (acid reflux, nonacid reflux [NAR], or no abnormal reflux). Mean nocturnal baseline impedance (MNBI) were measured 3 cm (distal esophagus) and 17 cm (proximal esophagus) above the lower esophageal sphincter. Median distal esophageal MNBI was lower in the acid reflux group (1244 Ω; 647–1969 Ω) than in the NAR (2586 Ω; 1368–3666 Ω) or no abnormal reflux groups (3082 Ω; 2495–4472 Ω; all *P* < .05). Distal MNBI were negatively correlated with DeMeester score and acid exposure time. Atypical symptoms were more frequently associated with NAR than typical symptoms (*P* < .01). Among patients with positive symptom-association probability (SAP) for NAR, median proximal MNBI tended to be lower in patients with typical symptoms (median, 3013 Ω; IQR, 2535–3410 Ω) than in those with atypical symptoms (median, 3386 Ω; IQR, 3044–3730 Ω, *P* = .05). Thus, atypical GERD symptoms were more likely to be associated with NAR. The mucosal integrity of the proximal esophagus might be relatively impaired in GERD patients with typical symptoms for NAR.

## 1 Introduction

Gastroesophageal reflux disease (GERD) is a condition in which gastroduodenal contents pass into the esophagus and cause troublesome symptoms and/or complications.^[1]^ GERD significantly diminishes the quality of life and increases healthcare costs. The typical symptoms of GERD include heartburn, acid regurgitation, and chest pain, whereas its atypical symptoms include abdominal discomfort, chronic cough, and asthma.^[2,3]^

The pathogenesis of GERD remains to be completely elucidated. Given the fact that GERD is a multifactorial disease, it is unlikely that its various symptoms will have the same pathogenesis. In patients with typical symptoms, reflux episodes damage the esophageal mucosa, which is manifested as mucosal erosions or dilated intercellular spaces (DIS). These changes result in the direct activation of sensory nerve endings and lead to the perception of heartburn, acid regurgitation, and chest pain.^[4]^ The atypical symptoms of GERD include both esophageal symptoms (eg, abdominal discomfort, nausea, and belching) and extraesophageal symptoms (eg, cough, asthma, and throat pain). Atypical esophageal symptoms may result from delayed gastric emptying, or proximal stomach expansion.^[5,6]^ In patients with extraesophageal symptoms, the reflux of gastric contents directly irritates the tissues of the laryngopharynx and tracheobronchial tree, or stimulates an esophageal-bronchial neural cough reflex.^[3]^ Both reflux episodes and esophageal mucosal barrier play a crucial role in the pathogenesis of GERD. We, therefore, compared the 2 between patients with typical and atypical GERD symptoms in this study.

Twenty-four-hour esophageal multichannel intraluminal impedance and pH (MII-pH) monitoring has been reported to be highly sensitive for the detection of all types of reflux events within the esophagus.^[7]^ Moreover, it is superior to conventional 24-h pH monitoring in distinguishing between acid reflux and nonacid reflux (NAR), and detecting correlations between reflux events and symptoms. In addition, MII-pH parameters are strongly associated with the presence of esophageal mucosal damage. Most studies have shown that baseline impedance levels (BILs) are lower in patients with GERD than in healthy individuals or those with functional heartburn.^[8–11]^ A negative correlation has been reported between BILs and intercellular spaces in the esophagus.^[8]^ However, few studies have compared the typical and atypical symptoms of GERD by using esophageal MII-pH monitoring.

We hypothesized that a relationship existed between reflux episodes and esophageal mucosal integrity (judged using BILs), and that both of these parameters differed between patients with typical and atypical GERD symptoms. Thus, our study aimed to clarify the relationship between BILs and reflux episodes, and compare reflux episodes and BILs between patients with typical and atypical symptoms of GERD.

## 2 Materials and methods

### 2.1 Subject selection and study groups

We retrospectively enrolled 110 patients who underwent investigations for the detection of GERD at the Gastrointestinal Motility Center of the First Affiliated Hospital of Nanjing Medical University, China, between January 2013 and June 2016. Patients were included in the study if they were 18 years or older in age and had experienced GERD symptoms at least 2 times a week lasting 6 months. Their Reflux Disease Questionnaire (RDQ) scores were not less than 12. Patients were excluded if they were found to have a tumor, peptic ulcer, or any mucosal abnormality other than esophagitis and superficial gastritis on endoscopy; a history of gastrointestinal surgery; severe organ dysfunction; or primary or secondary severe esophageal motility disorders (eg, achalasia, nutcracker esophagus, scleroderma, and diabetes mellitus).^[12]^ The study protocol was approved by the ethics committee of the First Affiliated Hospital of Nanjing Medical University. All patients provided informed consent before the start of the investigation.

### 2.2 Esophageal MII-pH monitoring

All subjects underwent MII-pH monitoring off-therapy, that is, proton pump inhibitors (PPIs), H_2_-receptor antagonists, and drugs influencing esophageal motor function were discontinued at least 14 days prior to the test. Esophageal intraluminal impedance and pH values were measured using an ambulatory MII-pH monitoring system (Given Imaging, Duluth, GA). A 2.3 mm MII-pH catheter was passed transnasally. The esophageal pH sensor was placed 5 cm above the lower esophageal sphincter (LES), and 6 intraluminal impedance channels (z1, z2, z3, z4, z5, and z6) were placed at 17, 15, 9, 7, 5, and 3 cm above the LES, respectively. The MII-pH parameters were measured automatically by the software of the monitoring system (Given Imaging). During the 24-h MII-pH test, meal timings, changes in body position, and timing of any symptoms were recorded by pressing the button on data recorder. These data were downloaded into a personal computer and automatically analyzed by the monitoring-system software. Above procedure was conventional routine according to reference and instructions of esophageal MII-pH monitoring.^[7,8]^ The following impedance and pH data were analyzed in this study: DeMeester score and acid exposure time (AET), number and type of reflux episodes, symptom-association probability (SAP), and BILs.

### 2.3 Gastroesophageal reflux parameters

As mentioned earlier, the impedance and pH data were used to determine the DeMeester score, 24-h AET, and the number and type of reflux episodes. AET was expressed as percentage, and defined as the total time for which the pH in the distal esophagus was below 4 divided by the total duration of MII-pH monitoring. A DeMeester score of more than 14.7 or an AET of more than 4.2% was considered to indicate abnormal distal esophageal acid exposure. Liquid refluxes were defined as acidic or nonacidic (including weakly acidic and weakly alkaline) refluxes, according to previously reported criteria.^[7]^ The total number of reflux episodes was noted (normal value, ≤73 in 24 hours).

The patients were divided into 3 groups: acid reflux group, patients with abnormal distal esophageal acid exposure (pH+); NAR group, patients with normal distal esophageal acid exposure but abnormal number of reflux events (MII+/pH−); and nonreflux group, patients with normal distal esophageal acid exposure and number of reflux events (MII−/pH−).

### 2.4 Symptom-reflux association analysis

Patients were categorized as having typical (regurgitation, heartburn, or chest pain) and/or atypical symptoms (belching, abdominal discomfort, hiccups, cough, nausea, or throat discomfort) on the day of MII-pH monitoring, as reported previously.^[13]^ The SAP for typical and atypical esophageal symptoms was considered positive if it exceeded 95%. We separated the typical and atypical symptoms associated with acid reflux from those associated with NAR.

An SAP >95% for acid reflux and negative for NAR was considered as a positive SAP for acid reflux only; an SAP >95% for NAR and negative for acid reflux was considered as a positive SAP for NAR only; and an SAP >95% for acid reflux and >95% for NAR was considered as a positive SAP for both acid reflux and NAR.

### 2.5 Mean nocturnal baseline impedance

When the esophageal lumen is empty, 2 consecutive sensors located at MII-pH catheter are in contact with the esophageal mucosa that has BILs. Mean nocturnal baseline impedance (MNBI) was assessed from the channels at 3 cm (distal esophageal BILs) and 17 cm (proximal esophageal BILs) above the LES. As previously described,^[14,15]^ MNBI (expressed in ohms) was measured during the overnight rest, at 3 time points (around 1.00, 2.00, and 3.00 a.m.), for a 10-minute period at around each time point, avoiding refluxes and swallows. The MNBI was calculated from the 3 impedance levels.

### 2.6 Statistical analysis

The Kolmogorov-Smirnov test was performed to assess the normality of the data. The data was expressed as mean ± SD or medians with interquartile ranges (IQRs: 25th–75th percentile) according to the normality of the data. Because the data were not normally distributed, the number of reflux episodes and BILs were expressed as medians with interquartile ranges (IQRs: 25th–75th percentile). Continuous variables were compared using the Kruskal-Wallis and/or Mann-Whitney *U* tests. Categorical variables were compared using the χ^2^ test or Fisher exact test depending on the sample size. The correlation of BILs with DeMeester score and AET was determined using the Pearson test. A *P* value of <.05 was considered statistically significant. All data were analyzed using SPSS (version 20; IBM Corp., Armonk, NY) and Prism software (version 5; Graph Pad, San Diego, CA).

## 3 Results

### 3.1 Demographics and clinical characteristics

A total of 110 subjects (54 men and 56 female) with a mean age of 51 ± 14 years (range, 20–80 years) were enrolled in our study. A total of 34 patients had erosive esophageal mucosa, 76 patients had normal esophageal mucosa on endoscopy. After MII-pH monitoring, 34 (31%) patients were assigned to the acid reflux group, 44 (40%) patients were assigned to the NAR group, and 32 (29%) subjects were included in the nonreflux group. Among the 78 patients with reflux, a total of 5962 reflux events were recorded, including 2025 (34%) episodes of acid reflux and 3937 (66%) episodes of NAR. The median number of total reflux episodes per patient was 45 (IQR, 22–72), 15 (4–28) in the acid reflux group, and 25 (13–45) in the NAR group.

Eighty-two (75%) patients recorded symptoms during the monitoring period, and 28 (25%) patients had no symptoms during this period. The 82 symptomatic patients recorded a total of 135 GERD symptoms, including 62 typical and 73 atypical symptoms. These symptoms (Table 1) consisted of the following: heartburn, 25 (23%) patients; regurgitation, 22 (20%) patients; belching, 22 (20%) patients; cough, 21 (19.1%) patients; chest pain, 15 (13.6%) patients; nausea, 11 (10%) patients; abdominal discomfort, 10 (9.1%) patients; hiccups, 7 (6.4%) patients; and throat discomfort, 2 (1.8%) patients.

**Table 1:**
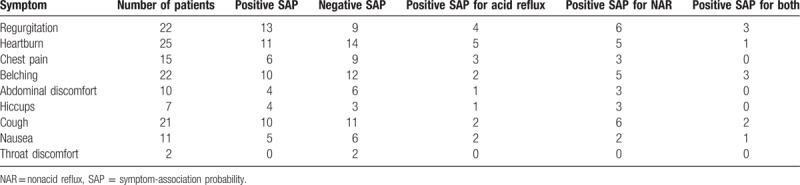
Symptom analysis using SAP.

### 3.2 Correlation between NMBI and reflux episodes

**Figure 1:**
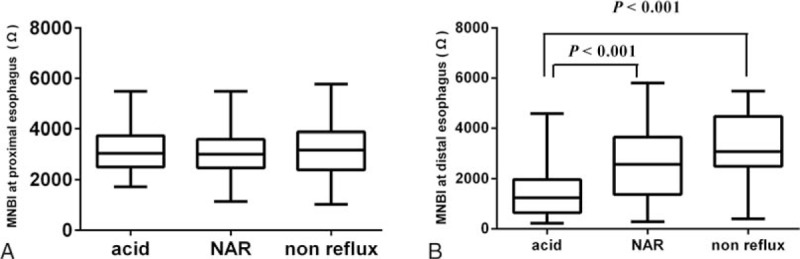
MNBI at the distal and proximal esophagus among different reflux groups. (A) Proximal esophageal MNBI do not differ among the study groups (*P* > .05). (B) Distal MNBI are lower in patients with acid reflux than in patients with NAR and nonreflux subjects (all *P* < .05). No difference in MNBI is present between patients with NAR and nonreflux subjects (*P* > .05). MNBI = mean nocturnal baseline impedance, NAR = nonacid reflux.

**Figure 2:**
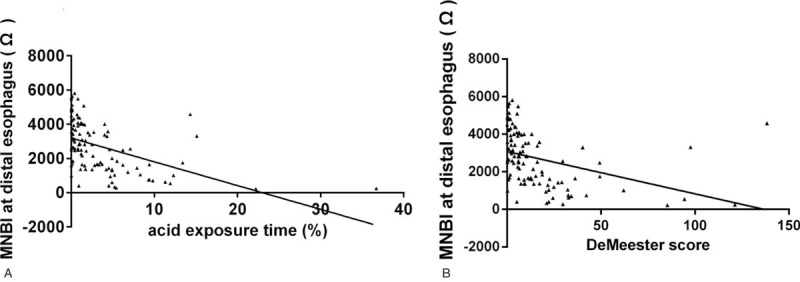
Distal MNBI are negatively correlated with (A) AET (*r* = −0.48, *P* < .01) and (B) DeMeester score (*r* = −0.37, *P* < .01). AET = acid exposure time, MNBI = mean nocturnal baseline impedance.

The median distal esophageal MNBI was significantly lower in the acid reflux group (1244 Ω; IQR, 647–1969 Ω) than in the NAR group (2586 Ω; IQR, 1368–3666 Ω) and nonreflux group (3082 Ω; IQR, 2495–4472 Ω, all *P* < .001; Fig. 1). Although the distal esophageal MNBI was lower in the NAR group than in the nonreflux group, no significant difference was found between the 2 groups (*P* = .78). We found that the distal esophageal MNBI was inversely correlated with the AET (*r* = −0.48, *P* < .001) and DeMeester score (*r* = −0.37, *P* < .001; Fig. 2).

The proximal esophageal MNBI did not differ among the acid reflux group (median, 3046 Ω; IQR, 2512–3471 Ω), NAR group (median, 3011 Ω; IQR, 2474–3599 Ω), and nonreflux group (median, 3177 Ω; IQR, 2395–3880 Ω; *P* = .87).

### 3.3 Correlation between symptom-reflux association and reflux episodes

**Figure 3:**
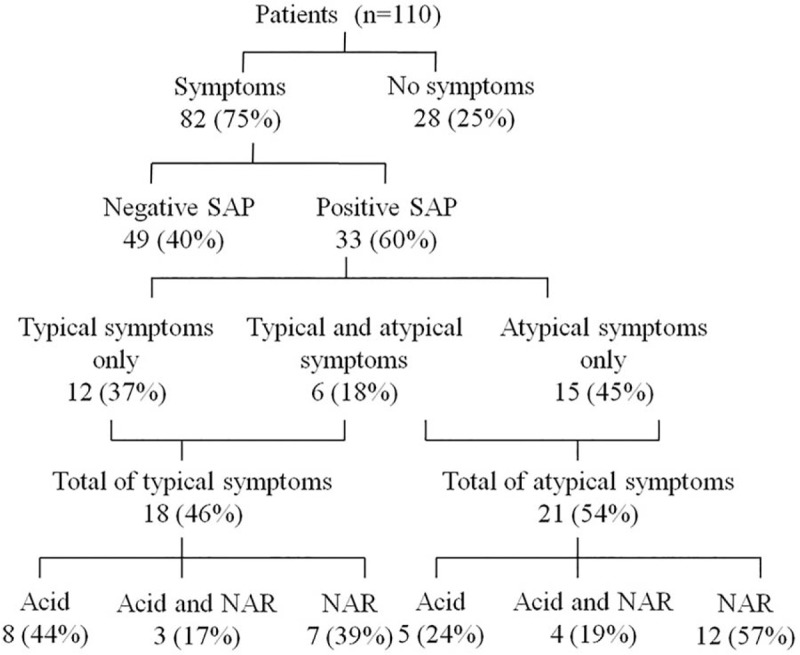
Relationship among typical and atypical symptoms of GERD, and reflux types. GERD = gastroesophageal reflux disease, NAR = nonacid reflux, SAP = symptom-association probability.

We further separated the 33 patients with positive SAP into those with typical symptoms and those with atypical symptoms (Fig. 3). Among these patients, 12 (37%) had typical symptoms only, 15 (45%) had atypical symptoms only, and 6 (18%) had both typical and atypical symptoms. The positive SAP was related to acid reflux only in 11 (33%) patients, to NAR only in 17 (52%) patients, and to both acid reflux and NAR in 5 (15%) patients.

Among the patients with positive SAP and typical symptoms, 8 (44%) had a positive SAP for acid reflux, 7 (39%) had a positive SAP for NAR, and 3 (17%) had a positive SAP for both reflux types. Among the patients with positive SAP and atypical symptoms, 5 (24%) had a positive SAP for acid reflux, 12 (57%) had a positive SAP for NAR, and 4 (19%) for both reflux types. Compared with typical symptoms, atypical symptoms were more likely to be related to NAR (*χ*^*2*^ = 6.4, *P* = .01).

### 3.4 Correlation between symptom-reflux association and MNBI

**Figure 4:**
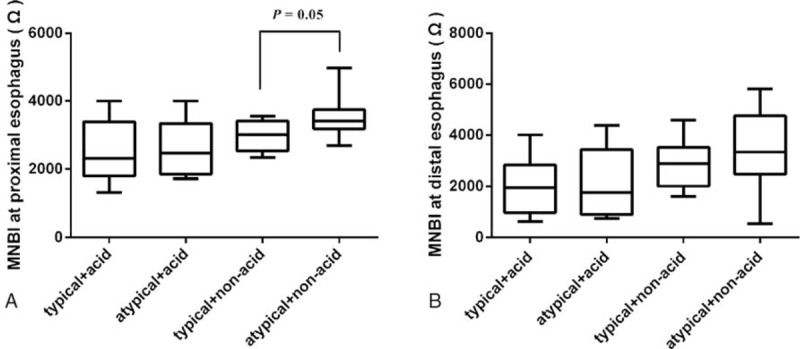
Correlation between symptom-reflux association and MNBI. (A) A trend of lower proximal MNBI is seen in patients with typical symptoms and positive SAP for NAR compared with patients with atypical symptoms and positive SAP for NAR (*P* = .05). (B) No differences in distal MNBI are seen between patients with typical and atypical symptoms with positive SAPs for acid reflux, and between patients with typical and atypical symptoms with positive SAPs for NAR (*P* > .05). MNBI = mean nocturnal baseline impedance, NAR = nonacid reflux, SAP = symptom-association probability.

Among patients with positive SAP for NAR, proximal MNBI tended to be lower in those with typical symptoms (median, 3013 Ω; IQR, 2535–3410 Ω) than in those with atypical symptoms (median, 3386 Ω; IQR, 3044–3730 Ω, *P* = .05). However, no significant difference in proximal MNBI was detected between patients with positive SAP for acid reflux and typical or atypical symptoms (median, 2318 Ω; IQR, 1795–3390 Ω vs median, 2468 Ω; IQR, 1854–3344 Ω; *P* = .86; Fig. 4A).

In addition, distal MNBI did not differ between patients with positive SAP for acid reflux and typical (median, 1953 Ω; IQR, 977–2833 Ω) or atypical symptoms (median, 1757 Ω; IQR, 898–3429 Ω, *P* > .99), or between patients with positive SAPs for NAR and typical (median, 2878 Ω; IQR, 2011–3517 Ω) or atypical symptoms (median, 3335 Ω; IQR, 2470–4748 Ω, *P* = .42; Fig. 4B).

## 4 Discussion

This study was designed to evaluate the correlation of reflux events with esophageal mucosal integrity (as judged by MNBI) and to compare these parameters between patients with typical and atypical symptoms of GERD. The main findings of this study were as follows: distal MNBI at 3 cm above the LES were lower in the acid reflux group than in the NAR or nonreflux groups; DeMeester score and AET were inversely correlated with distal MNBI at 3 cm; compared with typical symptoms, atypical symptoms were more likely to be associated with NAR; and among patients with positive SAP for NAR, proximal MNBI tended to be lower in those with typical symptoms than in those with atypical symptoms.

Acid reflux has been shown to trigger cellular damage in the esophageal mucosa in both in vitro and in vivo studies.^[9,16,17]^ Consistent with this, we found that distal MNBI were lower among patients with acid reflux than that with NAR or nonreflux. Moreover, distal MNBI were correlated with abnormal distal esophageal acid exposure (as indicated by the AET and DeMeester scores). This observation is consistent with those of previous studies.^[9,17,18]^

Caviglia et al demonstrated using transmission electron microscopy (TEM) that patients with nonerosive reflux disease (NERD) may have DIS not only in the distal but also in the proximal esophageal mucosa.^[18]^ Farre et al (2010) showed using TEM that distal esophageal acidic perfusion induced changes in not only the “exposed” distal mucosa but also in the more proximal “nonexposed” mucosa in healthy subjects.^[19]^ In contrast, the present study found that proximal MNBI did not significantly differ among the acid reflux, NAR, and nonreflux groups. A previous similar study by Farre et al (2011) showed that the baseline impedance at 3 cm above the LES was lower in GERD patients (both NERD and erosive esophagitis patients) than in healthy subjects; however, the impedance at 7 cm above the LES did not differ between the 2 groups.^[9]^ Farre et al offer 2 possible explanations for this difference: “(1) ultrastructural changes in the esophageal mucosa observed on TEM may not be related to functional alterations in the epithelium measured by intraluminal impedance, and (2) baseline impedance measurements may not be able to detect small ultrastructural alterations in the mucosa.”^[9]^

We also assessed the association of temporal reflux types (acid or nonacid) with gastroesophageal reflux symptoms (typical or atypical). Compared with typical symptoms, atypical symptoms were more likely to be related to NAR. Sifrim et al also showed that among patients with positive SAP during MII-pH monitoring, 75% cases of chronic cough were associated with nonacidic or weakly acidic reflux.^[20]^ As atypical symptoms appear to be primarily associated with NAR, MII-pH monitoring may be more accurate than pH monitoring alone or PPI tests for the detection of GERD. Moreover, PPI therapy is likely to fail in GERD patients with atypical symptoms. In fact, several randomized controlled trials have found no significant difference between PPI and placebos in relieving the extraesophageal symptoms of GERD.^[21–23]^ Unfortunately, few pharmacological agents are available to treat NAR at present. Among the currently available drugs,^[5,24]^ γ-aminobutyric acid B receptor agonists, metabotropic glutamate receptor 5 antagonists and prokinetic agents have been shown to have an effect on NAR; however, the routine use of these agents is not recommended because of the limited benefits and high risk of side effects.^[25]^ Surgical and endoscopic interventions may also be useful for NAR.^[26]^

There is evidence that the proximal esophagus appears to be more sensitive to mechanical distension, electrical stimuli,^[27]^ and acid-evoked heartburn and chest pain (typical symptoms) than the distal esophagus.^[28]^ A recent study found that proximal mucosal afferent nerves were in a more superficial location than distal nerves, and this may be the anatomical basis of enhanced sensitivity to reflux-evoked symptoms in the proximal esophagus.^[29]^ In addition, as noxious stimuli are more likely to gain access to sensory nerve endings through DIS, patients with impaired esophageal mucosa are more vulnerable to symptomatic perception.^[10,11,30,31]^ Therefore, we speculated whether mucosal integrity of esophagus, especially proximal esophagus, were difference between GERD patients with typical and atypical symptoms. In the present study, we characterized the MNBI in GERD patients with typical and atypical symptoms. Given the impact of acid on MNBI, we stratified typical and atypical symptoms according to the type of reflux (acid reflux or NAR). Interestingly, we only observed a trend of lower proximal MNBI in patients with typical symptoms associated with NAR than in patients with atypical symptoms associated with NAR. It is worth noting that when we divided the patients into 4 groups based on typical versus atypical symptoms and positive SAP for acid reflux versus NAR, the number of patients in each group was small. Thus, our results needed to be checked up with a large-scale cohort.

The limitations of our study are as follows: Our assessment of symptom perception was limited to the MII-pH monitoring period. There was a lack of healthy volunteers. However, patients with normal MII-pH parameters were selected as controls. We did not assess DIS by histological biopsy in the patients, as MNBI is a good marker for esophageal mucosal integrity.

In conclusion, distal MNBI are related to esophageal acid exposure, and were usually decreased in patients with abnormal distal esophageal acid exposure. This suggests that MNBI in the distal esophagus could be a marker for acid-induced alterations of the esophageal mucosa. In addition, GERD patients with atypical symptoms more frequently had NAR and a trend of higher proximal MNBI than patients with typical symptoms. These results indicated that there exist some differences in pathogenic mechanisms between GERD patients with typical and atypical symptoms. In future, the management of these 2 types of patients needs to be set up individually based on the clinical presentation and results of MII-pH monitoring.

## Acknowledgments

We are thankful to staff of the Department of Gastroenterology of the First Affiliated Hospital of Nanjing Medical University, China, for making summary results publicly available. We would like to give special thanks to Prof Hongjie Zhang.
